# Novel Longitudinal and Propensity Score Matched Analysis of Hands-On Cooking and Nutrition Education versus Traditional Clinical Education among 627 Medical Students

**DOI:** 10.1155/2015/656780

**Published:** 2015-09-08

**Authors:** Dominique J. Monlezun, Benjamin Leong, Esther Joo, Andrew G. Birkhead, Leah Sarris, Timothy S. Harlan

**Affiliations:** ^1^The Goldring Center for Culinary Medicine, Tulane University, New Orleans, LA 70119, USA; ^2^Tulane University School of Public Health & Tropical Medicine, New Orleans, LA 70112, USA; ^3^Long Beach Memorial Medical Center, Long Beach, CA 90806, USA; ^4^Harbor-University of California Los Angeles Medical Center, Torrance, CA 90502, USA; ^5^University of Illinois at Chicago College of Medicine, Chicago, IL 60612, USA

## Abstract

*Background*. Physicians are inadequately equipped to respond to the global obesity and nutrition-associated chronic disease epidemics. We investigated superiority of simulation-based medical education with deliberate practice (SBME-DP) hands-on cooking and nutrition elective in a medical school-based teaching kitchen versus traditional clinical education for medical students.* Materials and Methods*. A 59-question panel survey was distributed to an entire medical school twice annually from September 2012 to May 2014. Student diet and attitudes and competencies (DACs) counseling patients on nutrition were compared using conditional multivariate logistic regression, propensity score-weighted, and longitudinal panel analyses. Inverse-variance weighted meta-analysis (IVWM) was used for planned subgroup analysis by year and treatment estimates across the three methods.* Results*. Of the available 954 students, 65.72% (*n* = 627) unique students were followed to produce 963 responses. 11.32% (*n* = 109) of responses were from 84 subjects who participated in the elective. SBME-DP versus traditional education significantly improved fruit and vegetable diet (OR = 1.38, 95% CI: 1.07–1.79, *p* = 0.013) and attitudes (OR = 1.81, 95% CI: 1.40–2.35, *p* < 0.001) and competencies (OR = 1.72, 95% CI: 1.54–1.92, *p* < 0.001).* Conclusions*. This study reports for the first time superiority longitudinally for SBME-DP style nutrition education for medical students which has since expanded to 13 schools.

## 1. Introduction

The rising global obesity and nutrition-related chronic disease epidemics challenge medical education [1]. Obesity is a modifiable risk factor for three of the top four American mortality causes [2, 3], and its associated healthcare expenditures have doubled in just 10 years to $147 billion annually [4]. However, only half of primary care physicians regularly track body mass index (BMI) or provide nutrition education for their patients [5]. One potential cause of this deficit is insufficient early training [6]. Only two out of five American medical schools require the minimum 25 hours of nutrition education recommended by the National Academy of Sciences [7], leaving 71% of recently graduated medical students to report that they had been inadequately trained in nutrition counseling [8].

Previous pilot studies have demonstrated efficacy of nutrition education interventions to address these challenges. Yet their generalizability is limited by a lack of control comparison [9–15], validated survey metrics [9, 10, 14, 16], multiyear longitudinal follow-up [9, 11, 12, 14–16], and large sample size [9–13, 15, 16]. Past studies [9–16] also fail to incorporate the most extensively supported diet for patients, the Mediterranean diet [17–20], and two of the emerging hallmarks of evidence-based medical education, simulation-based medical education with deliberate practice (SBME-DP) and comparative effectiveness research (CER) [10, 12–16]. Their reliance on traditional clinical education without both simulation and deliberate practice has been shown to be inferior for skill acquisition in mastery learning, compared to the experiential learning approach of SBME-DP [21]. Furthermore, they fall outside of the Institute of Medicine (IOM) 2009 CER recommendations for national research funding priorities [22]. These previous nutrition studies emphasize efficacy of their intervention rather than the CER focus on effectiveness comparing a new treatment to existing standards. They also have reduced or absent coverage of the two leading CER priorities of healthcare delivery systems and racial and ethnic disparities [22].

We therefore sought to investigate superiority of SBME-DP style hands-on cooking and nutrition education elective over traditional clinical education for preventive medicine in a large sample of medical students in New Orleans, Louisiana. This multiyear prospective observational cohort study, Cooking for Health Optimization with Patients- (CHOP-) Medical Students, compared effectiveness of traditional clinical education given to a control group of students to an additional elective of cooking and nutrition education provided to the treatment group by a novel medical school-based teaching kitchen based in a lower income food desert community.

## 2. Methods

### 2.1. Curriculum

The Goldring Center for Culinary Medicine (GCCM) at Tulane University School of Medicine created the elective as part of a longitudinal curriculum from prior evidence-based curricula [23, 24] for future and current physicians to improve their nutrition counseling for patients in a CER framework [25–27]. Through the service learning requirements, medical students are able to sharpen their skill acquisition in a simulated environment as they first learn nutrition counseling and then deliberately practice the competency topics by teaching patients through GCCM's community cooking classes [26]. GCCM's curriculum and accompanying survey adopt the curriculum topics and validated metrics of student dietary habits and attitudes and competencies educating patients on nutrition education (DACs) from previously published curricula [11–13, 15]. Programming begins with the 28-hour student elective in nutrition education as an eight-module (class) series. The elective is for first and second year medical students over eight weeks in a medical school-based teaching kitchen on the Mediterranean diet and validated competency topics in patient nutrition counseling. The programming continues into disease-specific interdisciplinary seminars (IDs) for third year medical students, and concludes with a four week long away rotation for fourth year medical students at Rhode Island Hospital and Johnson & Wales University. Student-led community cooking classes as service learning are available for all class years.

Each module in the problem-based learning student elective features premodule 30-minute videos on lecture content with premodule quizzes, 45 minutes of precooking classroom discussion on gaps in knowledge shown in quiz responses, 1.5 hours of hands-on cooking which illustrates clinical and pathophysiology points from the lecture material, and 45 minutes eating the prepared meal during discussion of national board-style questions drawing from module material. Any first year and second year medical student can voluntarily enroll in the elective, any third year student in the IDs, and any fourth year in the away rotation. Promotion for these opportunities is done with the GCCM student interest group advertising on school-wide emails, class Facebook groups, and on-campus flyers to ensure the entire student body is aware of these offerings.

### 2.2. Data

GCCM began conducting in September 2012 a 59-question panel survey for the entire Tulane School of Medicine population of 954 medical students in New Orleans, Louisiana, at the beginning and end of each academic year. Data points included DACs and demographics to allow tracking of individual students. Beginning with the second fall survey, eight supplemental competency topics from the existing literature were added to the original 17 to provide students with more targeted training in nutrition counseling for patients. This addition followed the first year-end curriculum improvements using focus groups, qualitative student assessments, and the expertise of the chef and physician GCCM directors.

Study inclusion criteria included being generated in the first two years of the survey, reporting either GCCM elective participation or nonparticipation, and having only one unique survey response per student per survey date (September 2012, April 2013, September 2013, and April 2014). Students were encouraged to complete all four surveys over two years for time comparisons by group (GCCM or control) and individual responses. The Tulane Institutional Review Board approved this study, and subjects documented informed consent by clicking on the online survey link following the study description. Subjects were informed only their deidentified responses would be analyzed by the study team, thus communicating their grades would not be affected by their participation or nonparticipation since the school administration did not have access to their responses.

**Figure 1:**
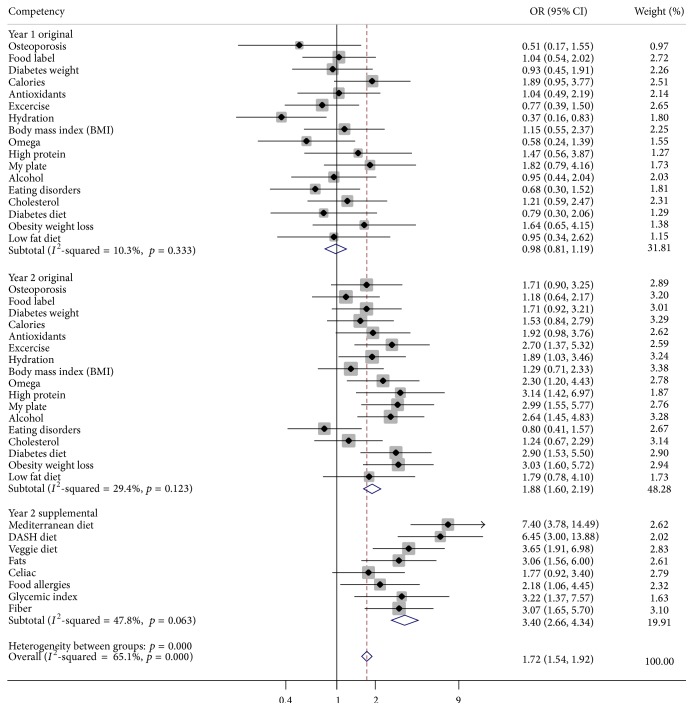
Pooled treatment effect of GCCM versus control on competencies using fully adjusted conditional multivariate logistic regression by year (*n* = 963 responses). Fully adjusted for gender, age, race, prior nutrition education, special diet, and clinical years' upperclassman. GCCM, Goldring Center for Culinary Medicine; OR, odds ratio; CI, confidence interval. Statistical significance defined by either two-tailed *p* value < 0.05 or ORs with 95% CIs not spanning 1.

Responses using a three-point Likert scale were utilized for attitudes and competencies in addition to a six-point scale for dietary habits that were both adopted from validated scales for appropriate investigation of the novel intervention [11–13, 15]. Although a five-point scale for attitudes and competencies was implemented in the second year of the study for improved assessment, the analysis had to translate that year's responses into the three points used in the first year for attitudes and competencies to aggregate the data. Responses were dichotomized to aid in interpretation for the following: agreement questions into strongly agree or not, dietary habits into daily intake or not, and competencies for totally proficient or not. The competency topics for patient nutrition counseling are listed in Figure 1.

### 2.3. Statistical Analysis

#### 2.3.1. Primary Analysis

Conditional multivariate logistic regression was used to investigate the association of GCCM elective participation compared to traditional clinical education (dependent variable) with DACs (independent variables). Conditional logistic regression was utilized to control for serial correlation (in which residuals could be correlated) in this time series data by matching subjects based on their survey date [28]. This method of multivariate logistic regression was chosen for the primary analysis due to its accepted application in controlling for observational trial selection bias by adjusting for known confounders and its noninferior performance versus the competing methods of propensity score (PS) analysis [29–31]. Conditional logistic regression was also chosen as the primary analysis as it does not require samples with similar treatment opportunity (compared to PS analysis) or repeat follow-ups from the same individual (compared to panel analysis). Entering medical student classes did not have the opportunity to participate in the GCCM education before the fall surveys and so PS analysis could only analyze those spring surveys after subjects had the opportunity to participate. In addition, not every student completed a follow-up survey and so panel analysis had to exclude those. Conditional multivariate logistic regression could therefore narrow the confidence intervals for greater precision with the treatment effect using a larger responder sample than PS and panel analyses. The following covariates were used for fully adjusted models due to their previously documented association with DACs: gender, age, race, prior nutrition education, special diet, and clinical years' upperclassman [9–16].

Given the lack of widely accepted validated psychometric endpoints for student competencies providing patients with nutrition counseling [9–16], we produced fully adjusted odds ratios for total proficiency in each competency topic, given GCCM education versus traditional clinical education. We then pooled these estimates and 95% confidence intervals (CIs) to one treatment effect estimate using an inverse-variance weighted meta-analysis (IVWM) that also included the test statistic for the pooled estimate being equal to one (Figure 1) [32]. This technique allowed responses to be missing at random and not completely at random in a longitudinal trial. Cochran's *Q* test and *I* ^2^ statistic were used to investigate heterogeneity of treatment effect across preplanned subgroup analysis by program year. A *p* value less than 0.10 indicated significant heterogeneity. This subgroup analysis sought to identify the DACs for GCCM to focus quality improvement efforts.

Sample size calculations based on the treatment effect of previously demonstrated nutrition education on medical trainees [9–16] indicated that 400 subject responses in total including 100 treated subject responses were needed to detect a 40% greater improvement in DACs for the treated subjects compared to the control with a power of 90%. To reduce the risk of response bias, we attempted to recruit all students in each class for each time period. This was logistically feasible given the fact that they were all geographically based at one medical school. Results were analyzed using STATA 12.0 (StataCorp LP, College Station, TX, USA). A *p* value < 0.05 was considered statistically significant.

#### 2.3.2. Secondary Analysis with Propensity Score and Panel Data

Preplanned secondary analysis featured PS-weighted logistic regression and longitudinal (panel) analysis with repeated measures adjusting for the same covariates from conditional multivariate logistic regression. Estimates were produced separately for DACs by both of these secondary methods and then compared to those produced by conditional multivariate logistic regression using IVWM. Cochran's *Q* test and *I* ^2^ statistic were used to determine if nonsignificant heterogeneity across the method subgroups indicated effect agreement.

PS analysis seeks to control for self-selection and unobserved heterogeneity from prebaseline differences between groups by statistically mimicking randomization [33–35]. This approach matches individuals from both treatment and control groups using observable differences to construct their probability of receiving the treatment. In PS-weighted regression models, the inverse probability of treatment weights (IPTW) was generated by the estimated propensity scores for outcome weighting. We based our PS-weighted regression analysis on the doubly robust approach by incorporating the same covariates in both the propensity score estimation model and outcome model [36]. This is meant to increase the probability of generating accurate treatment estimates on the outcome by increasing the chance that either the propensity score model or the outcome model is correctly specified. Fully adjusted models included the covariates from the conditional multivariate logistic regression analysis but using the PS as the weights.

This panel design allows comparison of not only the treatment group to the control group but also each group's individual outcomes to their baseline scores in a way that controls for such self-selection through fixed or random effect statistical models over time and between individuals [37]. Unobserved time-invariant individual traits like sociocultural and historical qualities can thus be controlled for using this panel analysis. A linear probability model of regression was fit to the panel data using either fixed effects by the within regression estimator or the random effects with the generalized least squares estimator to create an average of the within and between results with matrix weights. Panel analysis models were fully adjusted for using the covariates from conditional multivariate logistic regression. The Hausman test was used to determine whether to use the fixed or random effects model for each DAC [38].

For PS analysis, only the most recent spring survey per student was analyzed as per the requirements of this method, so students had sufficient study time to opt for participation in the treatment group. Panel analysis included all students who completed at least one subsequent survey following their first. The competencies for Mediterranean diet, DASH diet, vegetarian diet, dietary fats, food allergies, celiac disease, glycemic index, and fiber could not be investigated using this method because of the lower repeat response rates in the second spring survey after these new competencies were introduced in the second fall survey.

## 3. Results

### 3.1. Primary Analysis

Of the 954 Tulane students offered the survey over four semesters in two years, 65.72% (*n* = 627) unique students completed it to produce a total sample size of 963 responses meeting study criteria for primary analysis. Of this sample, 11.32% (*n* = 109) of responses were from 84 subjects who participated in the GCCM cooking and nutrition education elective to constitute the treatment group compared to the control group receiving traditional clinical education on nutrition in their school curriculum. In the treatment group, 68 (62.39%) were females, the mean age range was 25–29, 78 (72.22%) were white, 22 (20.37%) had prior nutrition education, 29 (26.85%) had a special diet, and 48 (44.04%) were 3rd or 4th year upperclassmen with clinical experience.

In fully adjusted conditional multivariate logistic regression, GCCM compared to traditional clinical education significantly increased the pooled odds of total proficiency in overall competencies by 72% (OR = 1.72, 95% CI: 1.54–1.92, *p* < 0.001) (Figure 1). This pooled estimate was generated from the fully adjusted odds ratios for each of the 25 competencies. In subgroup analysis by year, the improvements to the GCCM elective curriculum at the end of the first year nearly doubled the pooled treatment effect by the end of the second year compared to the first year curriculum (OR = 1.88, 95% CI: 1.60–2.20, *p* < 0.001 versus OR = 0.98, 95% CI: 0.81–1.19, *p* = 0.859) for the 17 original competencies and tripled it for the eight supplemental competencies (OR = 3.40, 95% CI: 2.66–4.34, *p* < 0.001). Cochran's *Q* test of *p* < 0.001 and *I* ^2^ statistic of 65.1% overall across the subgroups indicated that this improvement across the year subgroups was significant.

Figure 1 reports how GCCM versus traditional clinical education in fully adjusted models significantly increased the odds for students reporting attitudes that nutrition should be routine (OR = 1.88, 95% CI: 1.1821–2.9916, *p* = 0.008), specific nutrition advice can be efficacious (OR = 1.73, 95% CI: 1.10–2.7441, *p* = 0.019), and physician counseling can improve patients' diets (OR = 1.83, 95% CI: 1.1843–2.8349, *p* = 0.007) in addition to significantly increasing the odds of students' daily intake of nongreen vegetables (OR = 1.69, 95% CI: 1.08–2.65, *p* = 0.021). As with competencies, subgroup analysis by year revealed notable improvements in student attitudes and diet from the first year to the second year. Year one elective students compared to control students had improved odds of positive student attitudes about the importance and efficacy of nutrition counseling for patients' diets in addition to the students' own nongreen vegetable and fruit diet in the first year, though these were nonsignificant. But year two elective students compared to year two control students had significantly increased odds in all three attitudes: nutrition counseling should be routine (OR = 2.45, 95% CI: 1.3101–4.5734, *p* = 0.005), specific nutrition advice can be efficacious (OR = 2.37, 95% CI: 1.2433–4.5287, *p* = 0.009), and physicians can affect patients' diets (OR = 2.98, 95% CI: 1.6409–5.4263, *p* < 0.001). The improved GCCM curriculum in the second year also led to greater increases that were significant in daily dark green vegetable intake (OR = 2.11, 95% CI: 1.1546–3.8636, *p* = 0.015) and nongreen vegetable intake (OR = 1.92, 95% CI: 1.05–3.5171, *p* = 0.034).

### 3.2. Secondary Analysis

Of the 627 unique responders, 42.97% (*n* = 269) completed at least two surveys to allow longitudinal tracking of them in secondary panel analysis for a total sample size of 606 responses. For PS analysis, 626 responses met PS criteria. Using the inverse-variance weighted meta-analysis technique to compare DACs estimates from these methods with each other and from the primary analysis method, the fully adjusted treatment effect estimates were comparable across all three methods for diet and attitudes (Table 1). This was indicated by their nonsignificant heterogeneity for diet (*p* = 0.842) and attitudes (*p* = 0.693). There was significant heterogeneity for the treatment effect on competencies (*p* < 0.001). The significant pooled estimates using the fully adjusted odds ratios from all three methods were generated separately for diet (OR = 1.46, 95% CI: 1.22–1.75, *p* < 0.001), attitudes (OR = 1.87, 95% CI: 1.57–2.23, *p* < 0.001), and original competencies (OR = 1.90, 95% CI: 1.75–2.06, *p* < 0.001).

## 4. Discussion

In this large longitudinal CER study of medical students, we report superior effectiveness for a novel hands-on cooking and nutrition education over traditional clinical education improving student diets and their competencies and attitudes providing patients with nutrition counseling. Our study responds to the increasing calls from the medical profession for immediate action toward a longitudinal evidence-based nutrition curriculum [39]. Yet, importantly, our intervention diverges from other current leading education models [13, 40, 41] to demonstrate a promising model of today. GCCM's experiential hands-on cooking and nutrition curriculum differs from their older web-based or short-term programs as our medical students first simulate patient nutrition counseling in their own cooking and nutrition education classes before they then help lead classes for patient communities.

The uniqueness of the education intervention holds particular implications for CER-based improvement initiatives on the national stage with the ongoing Affordable Care Act (ACA) changes to the American healthcare system. ACA was intended to facilitate higher quality and lower cost healthcare through improved preventive medicine, within health systems integrating various healthcare utilization sources ranging from hospitals to community clinics to grocery stores [42]. The social determinants of health and health equity being explored by the World Health Organization along with other global institutions [43] further emphasize the not only national but also international implications of such a study as ours seeking optimization of the marriage between preventive medicine and healthcare. This study suggests how GCCM education may be a flawed but potentially promising approach toward these goals by training future physicians in preventive medicine. Over the past three years GCCM including its elective-trained students provided over 3,000 class hours for 409 community members increasingly from food desert and low-income areas. GCCM recently demonstrated through a randomized controlled trial of patients with type II diabetes that these scalable and sustainable classes can produce statistically significant improvements in diastolic blood pressure and cholesterol compared to the standard of care [25]. Ongoing GCCM CER studies are also analyzing curriculum impact on residents and practicing physicians to determine the long-term treatment effect on trainees through the medical education pipeline intro practice while also tracking their patient outcomes. Additionally, well-designed translational studies are required to confirm the above results.

In addition to the content of the intervention, we demonstrate possible advances in study methodology for preventive medicine through nutrition education among medical trainees. Prior to this, Lewis et al. [14] and Ray et al. [16] recently built on similar studies to make important contributions to such nutrition education methodology in this population. Lewis et al. applied a multicenter study design across 73 American residency programs (*n* = 322 trainees) to assess the association of online nutrition training modules and participant competencies in nutrition counseling. Ray et al. similarly used a cross-sectional study design to investigate the connection between their two-day workshop education and competencies among 100 students from 15 English medical schools but with further control group comparison and a validated metric (Knowledge, Attitudes, and Practices (KAP) score). We went further with our study assessing treatment effect of our unique intervention on 627 students over multiple years, comparing the outcomes from the treatment and the control groups using metrics adapted from multiple validated metrics before expanding analysis to 13 American medical schools in the subsequent study phase. Instead of utilizing, respectively, the simple ANOVA and score differences from Lewis et al. and Ray et al., we used three causal inference statistical techniques (conditional logistic regression, propensity score-weighted logistic regression, and panel regression) to adjust for multiple confounding factors that can predispose to selection bias (gender, age, race, prior nutrition education, special diet, and clinical years of medical education). The implication of this study's methodology therefore is detailing the closest known approximation at causal inference for research in nutrition education for medical trainees. Our well-powered study with longitudinal design uses an evidence-based intervention, rigorous statistical techniques reducing selection bias, and validated metrics to allow hopefully improved future studies in this subfield of preventive medicine filling the gap for evidence-based policy change in medical education.

Our findings thus respond to the larger global obesity and nutrition-related chronic disease epidemics by going beyond providing evidence of efficacy. We demonstrate greater effectiveness over the current education standard for building physician capacity for these epidemics. These results are also notable in that they demonstrate this effectiveness by seeking to robustly control for selection bias, as reported by similar estimates of the treatment effect across multiple rigorous statistical methods. Since randomization in medical education is logistically problematic, these comparable results across methods may suggest a sound analysis methodology that seeks to account for observed and unobserved baseline differences between treatment and control groups. The significant heterogeneity though for treatment effect estimates for competencies highlights importantly the need for validated metrics of student competencies in nutrition counseling and expanded sample sizes assessing them. But since the meta-analysis techniques used to create pooled estimates across methods are calculated with the variance of each estimate produced from the three methods, a larger sample size such as ours should decrease the variance for competency estimates. Aside from study methodology advances, the significant jump in DAC improvements across years suggests quality improvement advances. This finding indicates that real-time curriculum optimization based on student input and multidisciplinary collaboration between medical and culinary fields may offer a competitive model of team-based enhancements to medical education.

Our results should still be interpreted cautiously in the context of our limitations, which include the single-site design and incomplete follow-up that may be associated with the small but still notable heterogeneity in treatment effect estimates for competencies across the three statistical methods. The nonrandomized study design and attrition rate could add to selection bias, as students could exhibit nonrandom selection into the voluntary treatment group and follow-up tracking their outcomes. True treatment effect estimates may also be biased due to the lack of standardized curricula, metrics, analytic methods, and outcomes in nutrition education for medical professionals within the preventive medicine literature. The paucity of related studies and the varying quality of those in existence likely contribute to the absence of systematic reviews and meta-analyses to address these issues. We sought to reduce the impact of these limitations on the study by developing the elective curriculum with explicit reference to the existing evidence from previous studies, utilizing a longitudinal study design, recruiting a large sample, and applying rigorous statistical methods for causal inference. Finally, study limitations also include operator bias for the treatment as different instructors taught the GCCM elective, though the physician and chef directors for GCCM oversaw the training of instructors, their implementation of the curriculum, and regular performance reviews based on structures and unstructured focus group interviews with instructors and participants.

Even considering the above limitations, our novel study and treatment present compelling evidence that hands-on cooking and nutrition education compared to the standard of medical education can produce superior improvements in medical student training to provide patients with nutrition counseling. Expansion of CHOP-Medical Students as a longitudinal multisite phase II trial is underway with 13 medical schools for a nationally representative sample investigating the GCCM elective as a scalable and sustainable education model for the next generation of profound healthcare challenges in preventive medicine.

## Practice Points

The practice points are as follows:

(i) This is the first known large longitudinal comparative effectiveness study comparing hands-on cooking and nutrition education versus traditional clinical education for medical students' competencies in patient nutrition counseling.
(ii) Simulation-based medical education with deliberate practice (SBME-DP) hands-on education versus traditional education significantly increased the pooled odds of total proficiency in overall competencies (OR = 1.72, 95% CI: 1.54–1.92, *p* < 0.001).
(iii) Conditional multivariate logistic regression, propensity score-weighted, and longitudinal panel analyses produced similar significant estimates of the treatment outperforming the control for improving students' diets and attitude and competencies in providing patients with nutrition counseling.

## Figures and Tables

**Table 1:** Comparative effectiveness of GCCM elective versus traditional clinical education across three methods with fully adjusted models^*∗*^.

Outcome	IVWM average OR (95% CI)	C-logit (*n* = 963) OR (95% CI)	PSM (*n* = 626) OR (95% CI)	Panel (*n* = 606) OR (95% CI)	*Q* test *p* value
Daily diet of fruits and vegetables.	1.46 (1.22–1.75)	1.38 (1.07–1.79)	1.54 (1.15–2.05)	1.55 (0.95–2.55)	0.842
Strong agreement on nutrition counseling.	1.87 (1.57–2.23)	1.81 (1.40–2.35)	1.80 (1.35–2.39)	2.19 (1.47–3.28)	0.693
Totally proficient in original competencies.	1.90 (1.75–2.06)	1.49 (1.32–1.68)	2.26 (1.98–2.57)	2.52 (2.06–3.08)	<0.001

^*∗*^Fully adjusted for gender, age, race, prior nutrition education, special diet, and clinical years' upperclassman. IVWM, inverse-variance weighted meta-analysis; C-logit, multivariate conditional logistic regression; PSM, propensity score matching; *Q* test, *Q* test for heterogeneity across methods. Statistical significance defined by *p* value <0.05.

## References

[1] Ng M., Marie N., Tom F. (2014). Global, regional, and national prevalence of overweight and obesity in children and adults during 1980–2013: a systematic analysis for the Global Burden of Disease Study 2013. *The Lancet*.

[2] Yoon P. W., Bastian B., Anderson R. N., Collins J. L., Jaffe H. W. (2014). Potentially preventable deaths from the five leading causes of death—United States, 2008–2010. *Morbidity and Mortality Weekly Report*.

[3] Jensen M. D., Ryan D. H., Apovian C. M. (2014). 2013 AHA/ACC/TOS guideline for the management of overweight and obesity in adults: a report of the American College of cardiology/American Heart Association task force on practice guidelines and the obesity society. *Circulation*.

[4] Finkelstein E. A., Trogdon J. G., Cohen J. W., Dietz W. (2009). Annual medical spending attributable to obesity: payer-and service-specific estimates. *Health Affairs*.

[5] Smith A. W., Borowski L. A., Liu B. (2011). U.S. primary care physicians' diet-, physical activity—, and weight-related care of adult patients. *American Journal of Preventive Medicine*.

[6] Lenders C. M., Deen D. D., Bistrian B. (2014). Residency and specialties training in nutrition: a call for action. *American Journal of Clinical Nutrition*.

[7] Adams K. M., Kohlmeier M., Zeisel S. H. (2010). Nutrition education in U.S. medical schools: latest update of a national survey. *Academic Medicine*.

[8] Frantz D. J., McClave S. A., Hurt R. T., Miller K., Martindale R. G. (2015). Cross-sectional study of U.S. interns' perceptions of clinical nutrition education. *Journal of Parenteral & Enteral Nutrition*.

[9] Afaghi A., Mohamadi A. A. H. A., Ziaee A., Sarchami R. (2012). Effect of an integrated case-based nutrition curriculum on medical education at Qazvin University of Medical Sciences, Iran. *Global Journal of Health Science*.

[10] Barss P., Grivna M., Al-Maskari F., Kershaw G. (2008). Strengthening public health medicine training for medical students: development and evaluation of a lifestyle curriculum. *Medical Teacher*.

[11] Conroy M. B., Delichatsios H. K., Hafler J. P., Rigotti N. A. (2004). Impact of a preventive medicine and nutrition curriculum for medical students. *American Journal of Preventive Medicine*.

[12] Kohlmeier M., McConathy W. J., Cooksey Lindell K., Zeisel S. H. (2003). Adapting the contents of computer-based instruction based on knowledge tests maintains effectiveness of nutrition education. *The American Journal of Clinical Nutrition*.

[13] Lebensohn P., Kligler B., Dodds S. (2012). Integrative medicine in residency education: developing competency through online curriculum training. *The Journal of Graduate Medical Education*.

[14] Lewis K. O., Frank G. R., Nagel R. (2014). Pediatric trainees' engagement in the online nutrition curriculum: preliminary results. *BMC Medical Education*.

[15] Schlair S., Hanley K., Gillespie C. (2012). How medical students' behaviors and attitudes affect the impact of a brief curriculum on nutrition counseling. *Journal of Nutrition Education and Behavior*.

[16] Ray S., Udumyan R., Rajput-Ray M. (2012). Evaluation of a novel nutrition education intervention for medical students from across England. *BMJ Open*.

[17] Estruch R., Ros E., Salas-Salvadó J. (2013). Primary prevention of cardiovascular disease with a Mediterranean diet. *The New England Journal of Medicine*.

[18] Schwingshackl L., Hoffmann G. (2014). Adherence to Mediterranean diet and risk of cancer: a systematic review and meta-analysis of observational studies. *International Journal of Cancer*.

[19] Ajala O., English P., Pinkney J. (2013). Systematic review and meta-analysis of different dietary approaches to the management of type 2 diabetes. *The American Journal of Clinical Nutrition*.

[20] Esposito K., Maiorino M. I., Bellastella G., Chiodini P., Panagiotakos D., Giugliano D. (2015). A journey into a Mediterranean diet and type 2 diabetes: a systematic review with meta-analyses. *BMJ Open*.

[21] McGaghie W. C., Issenberg S. B., Cohen E. R., Barsuk J. H., Wayne D. B. (2011). Does simulation-based medical education with deliberate practice yield better results than traditional clinical education? A meta-analytic comparative review of the evidence. *Academic Medicine*.

[22] Iglehart J. K. (2009). Prioritizing comparative-effectiveness research—IOM recommendations. *The New England Journal of Medicine*.

[23] (1997). Report of the American Medical Student Association's Nutrition Curriculum Project. Essentials of nutrition education in medical schools: a national consensus. *The American Journal of Clinical Nutrition*.

[24] Bhargava A. (2010). Preventive nutrition: the comprehensive guide for health professionals. *The Journal of the American Medical Association*.

[25] Monlezun D. J., Kasprowicz E., Tosh K. W. (2015). Medical school-based teaching kitchen improves HbA1c, blood pressure, and cholesterol for patients with type 2 diabetes: results from a novel randomized controlled trial. *Diabetes Research and Clinical Practice*.

[26] Birkhead A. G., Foote S., Monlezun D. J. (2014). Medical student-led community cooking classes. *American Journal of Preventive Medicine*.

[27] Leong B., Ren D., Monlezun D., Ly D., Sarris L., Harlan T. S. (2014). Teaching third and fourth year medical students how to cook: an innovative approach to training students in lifestyle modification for chronic disease management. *Medical Science Educator*.

[28] Hosmer D. W., Lemeshow S., Cook E. D. (2001). *Applied Logistic Regression, Second Edition: Book and Solutions Manual Set*.

[29] Kurth T., Walker A. M., Glynn R. J. (2006). Results of multivariable logistic regression, propensity matching, propensity adjustment, and propensity-based weighting under conditions of nonuniform effect. *American Journal of Epidemiology*.

[30] Wyss R., Ellis A. R., Brookhart M. A. (2014). The role of prediction modeling in propensity score estimation: an evaluation of logistic regression, bcart, and the covariate-balancing propensity score. *American Journal of Epidemiology*.

[31] Li L., Vollmer W. M., Butler M. G., Wu P., Kharbanda E. O., Wu A. C. (2014). A comparison of confounding adjustment methods for assessment of asthma controller medication effectiveness. *American Journal of Epidemiology*.

[32] Borenstein M., Hedges L. V., Higgins J. P. T., Rothstein H. R. (2011). *Introduction to Meta-Analysis*.

[33] Thoa N. T. M., Thanh N. X., Chuc N. T. K., Lindholm L. (2013). The impact of economic growth on health care utilization: a longitudinal study in rural Vietnam. *International Journal for Equity in Health*.

[34] Austin P. C. (2014). Double propensity-score adjustment: a solution to design bias or bias due to incomplete matching. *Statistical Methods in Medical Research*.

[35] Austin P. C., Small D. S. (2014). The use of bootstrapping when using propensity-score matching without replacement: a simulation study. *Statistics in Medicine*.

[36] Shinozaki T., Matsuyama Y. (2015). Doubly robust estimation of standardized risk difference and ratio in the exposed population. *Epidemiology*.

[37] Gunasekara F. I., Richardson K., Carter K., Blakely T. (2014). Fixed effects analysis of repeated measures data. *International Journal of Epidemiology*.

[38] Dieleman J. L., Templin T. (2014). Random-effects, fixed-effects and the within-between specification for clustered data in observational health studies: a simulation study. *PLoS ONE*.

[39] Devries S., Dalen J. E., Eisenberg D. M. (2014). A deficiency of nutrition education in medical training. *The American Journal of Medicine*.

[40] Adams K. M., Kohlmeier M., Powell M., Zeisel S. H. (2010). Nutrition in medicine: nutrition education for medical students and residents. *Nutrition in Clinical Practice*.

[41] Eisenberg D. M., Myrdal Miller A., McManus K., Burgess J., Bernstein A. M. (2013). Enhancing medical education to address obesity: ‘See one. Taste one. Cook one. Teach one.’. *JAMA Internal Medicine*.

[42] Blumenthal D., Abrams M., Nuzum R. (2015). The affordable care act at 5 years. *The New England Journal of Medicine*.

[43] Marmot M., Allen J. J. (2014). Social determinants of health equity. *American Journal of Public Health*.

